# Downregulation of ADAMTS8 by DNA Hypermethylation in Gastric Cancer and Its Clinical Significance

**DOI:** 10.1155/2016/5083841

**Published:** 2016-07-14

**Authors:** Jing Chen, Jiakui Zhang, Xin Li, Chundong Zhang, Hongbin Zhang, Junzhe Jin, Dongqiu Dai

**Affiliations:** Department of Gastrointestinal Surgery, The Fourth Affiliated Hospital, China Medical University, No. 4 Chongshandong Road, Shenyang 110032, China

## Abstract

A disintegrin and metallopeptidase with thrombospondin motif type 8 (ADAMTS8), a member of the ADAMTS family, was discovered as a novel angiogenesis inhibitor. We analyzed the expression and methylation of ADAMTS8 in primary gastric tumors and gastric cancer cell lines. We also examined the relationship between ADAMTS8 expression and methylation and clinicopathologic features. The results showed that the significant downregulation of ADAMTS8 mRNA expression was observed in gastric cancer cell lines and tissues, and its expression was related to invasive depth and lymph node metastasis. CpG was hypermethylated in gastric cancer cell lines MKN45, MGC803, and BGC823, as well as primary gastric cancer specimens. ADAMTS8 mRNA expression was significantly lower in methylated primary gastric tumors. A significant association was found between ADAMTS8 methylation status and lymph node metastasis in primary gastric cancer. Moreover, ADAMTS8 expression was upregulated in the gastric cancer cell lines MGC803, BGC823, and MKN45 after treatment with 5-aza-2′-deoxycytidine. Thus, our results demonstrate that expression of ADAMTS8 mRNA is significantly decreased and DNA methylation is frequent in gastric cancer. ADAMTS8 hypermethylation is associated with decreased expression in gastric cancer and may play an important role in the invasion and metastasis of gastric cancer.

## 1. Introduction

Gastric cancer is one of the most common digestive malignancies worldwide, and more than 70% of new cases and deaths occur in developing countries [1]. Although the global incidence rate has declined in recent decades, this disease remains common in many regions, including China and Japan [2]. Many studies have demonstrated that epigenetic alterations, including DNA methylation and histone modifications, may result in the silencing of cancer-related genes. DNA methylation has become recognized as the most common epigenetic event in human cancers and plays a critical role in tumorigenesis. A number of tumor suppressor genes are silenced by hypermethylation in gastric cancer [3–5].

A disintegrin and metalloproteinases with thrombospondin motifs (ADAMTSs), a family of extracellular matrix metalloproteinase that contains 19 members, are similar to matrix metalloproteinases (MMPs) and ADAMs in structure and function [6]. ADAMTSs are secreted extracellular enzymes that have a compound domain organization comprising a signal peptide followed by a proregion of variable length, a metalloproteinase domain, a disintegrin-like domain, a central thrombospondin type 1 sequence repeat motif, and a cysteine-rich domain followed by a spacer region [7]. ADAMTS genes participate in a wide range of physiological processes including extracellular matrix (ECM) degradation, cell proliferation, apoptosis, migration, invasion, and angiogenesis [8–10] in a variety of diseases including thrombotic thrombocytopenic purpura [11, 12], osteoarthritis [13, 14], and cancer [9, 15, 16]. Recent studies have provided evidence of dysregulated ADAMTS expression in diverse types of cancers including gastric, colorectal, pancreatic, lung, esophageal, nasopharyngeal, and breast cancers [17–21]. ADAMTS8, also known as METH-2, is a member of the ADAMTS family and is originally identified as one of the antiangiogenic factors [22, 23]. It can inhibit VEGF-mediated angiogenesis in endothelial cells* in vitro* [22] as a secreted protease inhibits epidermal growth factor receptor (EGFR) signaling, resulting in decreased levels of phosphorylated MEK and ERK [24]. Moreover, the decrease in ADAMTS8 expression has been documented in some cancers [16, 20, 23, 25, 26] that ADAMTS8 shows high frequency of promoter methylation in brain, lung, and thyroid cancer [20, 23, 27] suggesting that the epigenetic silencing of ADAMTS8 may be involved in tumorigenesis. However, its tumor suppressive functions and underlying mechanisms in gastric cancer remain unknown. Thus, in the present study, we examined ADAMTS8 expression in gastric cancer cell lines and tissue samples and investigated the epigenetic mechanisms responsible for the decreased ADAMTS8 expression in gastric cancer.

## 2. Materials and Methods

### 2.1. Gastric Tissue Samples

A total of 66 paired nontumor tissues and tumor samples were obtained from gastric cancer patients who underwent a gastrectomy between January 2013 and December 2014, in the Fourth Affiliated Hospital of China Medical University. None of these patients had been treated with chemotherapy and radiotherapy before surgery. All tissue samples were divided into 2 blocks. One block was frozen in liquid nitrogen immediately after surgery and stored at −80°C and the other block was fixed in 5% formaldehyde solution, embedded in paraffin, and then cut into 5 *μ*m sections for HE staining and pathological examination. The nontumor tissues were at least 5 cm away from the tumor margin. All of the cases were pathologically confirmed. This study was approved by the Ethics Committee of Fourth Affiliated Hospital of China Medical University, and all of the patients signed an informed consent in this study.

### 2.2. Cell Lines and Culture

Four human gastric cancer cell lines, MGC803, MKN45, BGC823, and SGC7901, and the immortalized normal gastric cell line GES1 were cultured in RPMI 1640 (Gibco BRL, Grand Island, NY, USA) supplemented with 10% fetal bovine serum (Gibco) and incubated in a humidified atmosphere of 5% CO_2_ at 37°C. MGC803, MKN45, BGC823, and SGC7901 cells were obtained from the Institute of Biochemistry and Cell Biology, Chinese Academy of Sciences (Shanghai, China). The GES1 cell line was obtained from the Oncology Institute of China Medical University.

### 2.3. RNA Extraction and Quantitative Polymerase Chain Reaction (qPCR)

Total RNA from cultured cells, fresh frozen gastric cancer tissues, and corresponding nontumor tissues were isolated using TRIzol reagent (Invitrogen, Carlsbad, CA, USA) and converted to cDNA using an Expand Reverse Transcriptase Kit (Takara, Japan). qPCR was performed with the following program: 95°C for 30 s, 35 cycles of 95°C for 5 s, and 60°C for 30 s. The PCR mix was composed of 12.5 *μ*L SYBR Green (Takara), 1 *μ*L of each primer, 2 *μ*L cDNA, and 8.5 *μ*L diethylpyrocarbonate- (DEPC-) treated water. The primers for the human ADAMTS8 gene were 5′-AAC AAA AGC TGC TCC GTG AT-3′ (forward) and 5′-TCT GGT TCA GGT GGA CGA AC-3′ (reverse), which generated a 175-base pair (bp) fragment [23]. GAPDH was amplified as an internal control using the following primers: 5′-GAA GGT CGG AGT CAA CGG AT-3′ (forward) and 5′-CCT GGA AGA TGG TGA TGG GAT-3′ (reverse), which generated a 224 bp fragment. DEPC-treated water was used as the negative control in every PCR. The ADAMTS8 level was normalized to the level of GAPDH mRNA [28].

### 2.4. Methylation-Specific PCR (MSP) and Bisulfite Genomic Sequencing (BGS)

The genomic DNA was prepared from cell lines and tissues by the phenol/chloroform protocol and was modified by bisulfate treatment as previously described [29]. Briefly, 2 *μ*g genomic DNA was treated with NaOH (0.2 M) at 42°C for 30 min and denatured at 95°C for 5 min. Then, the DNA samples were cultured with sodium bisulfate and hydroquinone at 54°C for 16 h in the dark. The DNA was purified using a DNA cleanup system (Promega Corporation, Madison, WI, USA) followed by incubation with 0.3 M NaOH at 37°C for 15 min and precipitation with ammonium acetate and 100% ethanol at 20°C overnight. The next day, DNA was washed with 70% ethanol and dissolved in 15 *μ*L TE buffer. The MSP primer sequences used for the methylation reaction were 5′-CGG TAG TTT ATT CGG TGT TTT TC-3′ (forward) and 5′-CCT CTA ACT AAC GCA ACC CG-3′ (reverse), which generated a 164 bp fragment (from +367 to +530). The MSP primer sequences for the unmethylation reaction were 5′-TTG GTA GTT TAT TTG GTG TTT TTT G-3′ (forward) and 5′-CCC TCT AAC TAA CAC AAC CCA CT-3′ (reverse), which also generated a 166 bp fragment (from +366 to +531). PCR conditions were as follows: 94°C for 5 min, 94°C for 30 s, 40 cycles of 72°C for 45 s, 59°C for 30 s, 72°C for 30 s, and 72°C for 10 min. Human placental DNA treated* in vitro* with SssI methylase (New England Biolabs, Ipswich, MA, USA) and water served as positive and negative controls, respectively. The PCR products were separated by electrophoresis on a 2% agarose gel. For BGS, bisulfite-treated DNA was amplified for using BGS primers, and the PCR products were subcloned into the PCR4-TOPO vector. Five randomly chosen clones of each specimen were sequenced. The BGS primer sequences were 5′-AAA GGG TTA GTT TAG TAT GGT AGG G-3′ (forward) and 5′-ACA ACA ACA ACA ACA ACA ACA AC-3′ (reverse), which generated a 670 bp fragment containing 63 CpGs at the 5′-end region of ADAMTS8 (from +102 to +771).

### 2.5. Treatment of Cells with 5-Aza-2′-deoxycytidine

To assess reactivation of ADAMTS8 expression, four gastric cell lines were treated with 5 *μ*M 5-aza-2′-deoxycytidine (ADC; Sigma Chemical Co., St. Louis, MO, USA). MGC803, MKN45, BGC823, and SGC7901 were seeded at 5 × 10^5^ cells per well in 6-well culture plates and incubated in RPMI 1640 supplemented with 10% fetal bovine serum at a humidified atmosphere of 5% CO_2_ at 37°C. Treatment was performed over 3 days with daily media changes and addition of fresh ADC. Genomic DNA and total RNA were extracted from the cells before and after ADC treatment and were used for MSP and qPCR. In addition, MKN45 cells were treated with 2.5, 5, and 10 *μ*M ADC for 3 days, with daily media changes. After treatment, cells were harvested for RNA extraction and qPCR. The analysis was repeated three times.

### 2.6. Data Analysis

All statistical analyses were performed using SPSS version 16.0 software package. The nonparametric Mann-Whitney *U* test, Kruskal-Wallis test, and Wilcoxon signed-rank test were used to evaluate mRNA expression levels of ADAMTS8 in cell lines, tissue samples, and its relationship with clinicopathological factors. The *χ* ^2^ test was used to test the relationship between ADAMTS8 methylation and clinicopathological characteristics. Data are expressed as the mean ± standard deviation. A *P* value < 0.05 was considered statistically significant.

## 3. Results

### 3.1. ADAMTS8 mRNA Expression in Cell Lines and Gastric Tumors

**Figure 1:**
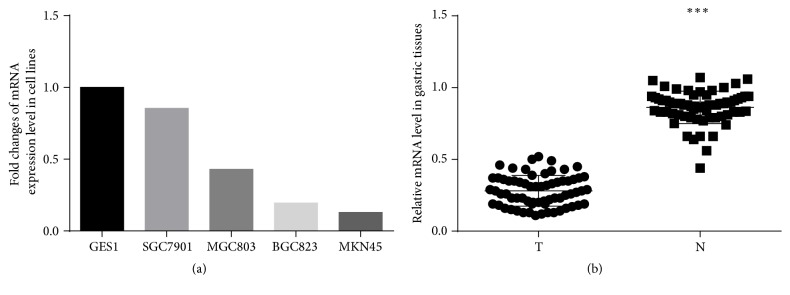
qPCR analysis of ADAMTS8 mRNA expression in cell lines and tissues. (a) ADAMTS8 mRNA expression level in GES1 and four gastric cancer cell lines. Fold change of ADAMTS8 expression was calculated relative to that of GES1 (1-fold as the control). (b) ADAMTS8 mRNA expression level in paired primary gastric tumors and corresponding nontumor tissues. T: primary gastric tumors; N: nontumor tissues (^*∗∗∗*^ *P* < 0.001).

qPCR was performed to evaluate the mRNA expression of ADAMTS8 in the tested cell lines. The results showed that ADAMTS8 mRNA expression was significantly lower in the gastric cancer cell lines SGC7901 (0.85-fold), MGC803 (0.43-fold), BGC823 (0.19-fold), and MKN45 (0.13-fold) compared to the normal gastric cell line GES1 (1-fold as the control) (Figure 1(a)). In addition, ADAMTS8 mRNA expression was analyzed in 66 paired primary gastric cancer specimens and corresponding nontumor tissues by qPCR. The results indicated that ADAMTS8 mRNA expression was significantly lower in the primary gastric cancer tissues than in their corresponding nontumor tissues (0.247 ± 0.076 versus 0.881 ± 0.098; *P* < 0.001; Figure 1(b)).

### 3.2. Aberrant Methylation Contributes to ADAMTS8 Downregulation in Cell Lines and Gastric Tumors

**Figure 2:**
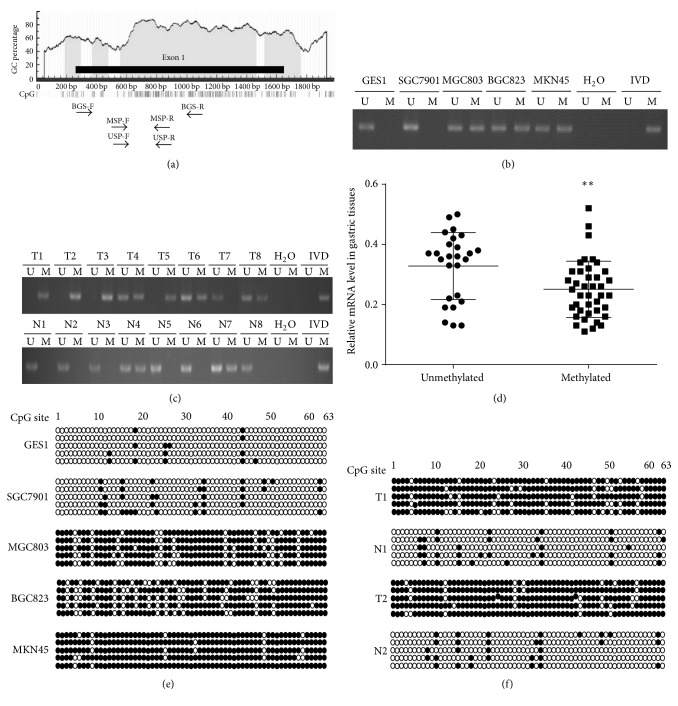
The ADAMTS8 methylation status in gastric cancer cells and gastric tumor samples. (a) Schematic diagram of CpG islands (CGI) in ADAMTS8. CGI across ADAMTS8 promoter and exon 1. The vertical bars indicate the location of each of the CpG sites and the thick line indicates the location of the MSP primers and BGS primers. (b) Methylation of ADAMTS8 in GES1 and four gastric cancer cell lines by MSP analysis. (c) Methylation of ADAMTS8 in paired primary gastric tumors and corresponding nontumor tissues by MSP analysis. U: unmethylated; M: methylated; IVD: positive control; H_2_O: negative control. (d) Association between ADAMTS8 mRNA expression level and ADAMTS8 methylation (^*∗∗*^ *P* < 0.01). (e) ADAMTS8 CpG methylation status in GES1 and four gastric cancer cells by BGS. (f) ADAMTS8 CpG methylation status in two matched primary gastric tumors and corresponding nontumor tissues by BGS.

Since the expression of ADAMTS8 was downregulated in gastric cancer, we examined whether the gene was silenced by hypermethylation. Analysis of ADAMTS8 DNA methylation status using the online database (http://www.urogene.org/) revealed a typical CGI across the promoter and exon 1 (Figure 2(a)). Accordingly, the DNA methylation status of ADAMTS8 in cell lines was assessed by MSP. The results confirmed in Figure 2(b) that ADAMTS8 was hypermethylated (one allele is methylated) in MKN45, partially methylated (both alleles are methylated) in MGC803 and BGC823, and unmethylated (neither allele is methylated) in SGC7901 and GES1 cells. These results were consistent with the qPCR analysis: the low-expressing cell line MKN45 exhibited hypermethylated CpGs, the intermediate-expressing cell lines MGC803 and BGC823 showed partially methylated CpGs, and the high-expressing cell lines SGC7901 and GES1 cells exhibited unhypermethylated CpGs. The methylation status of the ADAMTS8 was also examined in 66 primary gastric tumors and nontumor tissues by MSP (Figure 2(c)). The data showed that methylation of the ADAMTS8 was detected in 60.6% (40/66) of gastric tumor tissues, and 39.4% (26/66) were unmethylated. In contrast, DNA methylation was found in 40.9% (27/66) of nontumor tissues and unmethylation was found in 59.1% (39/66). Methylation of the ADAMTS8 gene was significantly higher in primary gastric tumors than in nontumor tissues (*P* = 0.024, Table 1). Furthermore, a strong correlation between methylation frequency and mRNA expression level was found in primary gastric tumors. ADAMTS8 mRNA expression was significantly lower in primary gastric tumors with DNA methylation of ADAMTS8 than those without DNA methylation (*P* < 0.001; Figure 2(d)).

We further analyzed ADAMTS8 methylation status in gastric cancer cell lines and two matched gastric tumors and nontumor tissues by BGS. As shown in Figure 2(e), the methylation rates of the ADAMTS8 gene in cell lines GES1, SGC7901, MGC803, BGC823, and MKN45 were 4.4%, 11.7%, 76.5%, 79.3%, and 93.3%, respectively. In addition, BGS revealed high levels of DNA methylation in gastric tumors compared with nontumor tissues (85.1% and 90.5% in gastric tumor tissue and 11.1% and 9.8% in nontumor tissues, resp.) (Figure 2(f)). These data are consistent with the MSP methylation results. Collectively, these results demonstrate that aberrant CpGs hypermethylation is common and is associated with decreased expression.

### 3.3. Effects of ADC on the mRNA Expression Levels and Methylation Status of ADAMTS8 in Gastric Cell Lines

**Figure 3:**
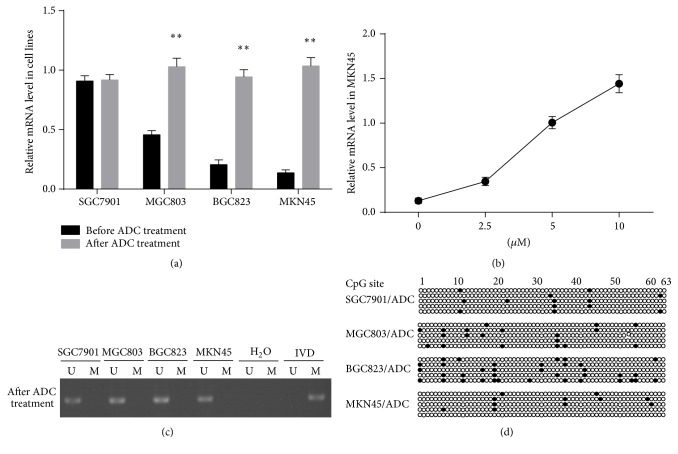
The ADAMTS8 mRNA expression and methylation status prior to and following treatment with ADC. (a) ADAMTS8 mRNA expression level in four gastric cancer cell lines prior to and following treatment with ADC (^*∗∗*^ *P* < 0.01). (b) ADAMTS8 mRNA expression level in MKN45 cell line prior to and following treatment with different concentrations of ADC. (c) Methylation of ADAMTS8 following treatment with ADC by MSP analysis. (d) ADAMTS8 CpG methylation status following treatment with ADC by BGS.

We treated four gastric cancer cell lines with demethylation agent ADC to inhibit DNA methylation and detected ADAMTS8 mRNA expression using qPCR analysis. ADC treatment significantly restored ADAMTS8 mRNA expression in MGC803 (1.030 ± 0.070 versus 0.457 ± 0.035; *P* < 0.01), BGC823 (0.944 ± 0.061 versus 0.206 ± 0.039; *P* < 0.01), and MKN45 (1.035 ± 0.071 versus 0.137 ± 0.025; *P* < 0.01) cell lines (Figure 3(a)). In addition, we treated the MKN45 cell line with low ADAMTS8 level for 3 days with 0, 2.5, 5, and 10 *μ*M ADC and found that ADAMTS8 mRNA were significantly upregulated after ADC treatment. Furthermore, we found that the effects of ADC on ADAMTS8 mRNA expression were dose-dependent, with higher doses yielding increased changes in ADAMTS8 mRNA expression (Figure 3(b)).

In addition, the effects of ADC treatment on ADAMTS8 DNA methylation status were assessed in the gastric cancer cell lines. The MSP results showed that treatment with ADC resulted in DNA demethylation in hypermethylated MGC803, BGC823, and MKN45 cells. In contrast, treatment with ADC had no significant effects on DNA methylation in unmethylated SGC7901 cells (Figure 3(c)). Using BGS, demethylation of ADAMTS8 was equally detected in MGC803, BGC823, and MKN45 cells after ADC treatment (Figure 3(d)). These results confirmed that ADC decreased the methylation levels and increased the demethylation levels of ADAMTS8 in gastric cancer.

### 3.4. Association of ADAMTS8 mRNA Expression with Clinicopathological Factors in Gastric Cancer

**Figure 4:**
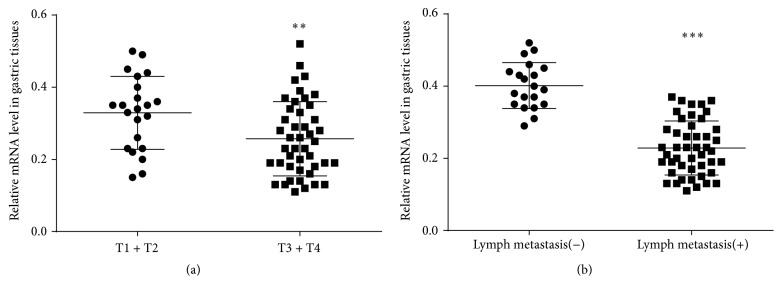
qPCR analysis of ADAMTS8 mRNA expression in gastric cancer patients. (a) ADAMTS8 mRNA expression level in patients with invasive depth T1 + T2 and those with invasive depth T3 + T4 (^*∗∗*^ *P* < 0.01). (b) ADAMTS8 mRNA expression level in patients with lymph node metastasis and those without lymph node metastasis (^*∗∗∗*^ *P* < 0.001).

Table 1 shows the relationship between ADAMTS8 mRNA expression and clinical characteristics in the 66 gastric cancer cases. There was a significant correlation between the mRNA expression level of ADAMTS8 and invasive depth (*P* = 0.009) as well as lymph node metastasis (*P* < 0.001). ADAMTS8 showed decreased mRNA expression in the primary tumors with T3 + T4 and lymph node metastasis. Otherwise, there was no association between ADAMTS8 mRNA expression and other clinicopathological factors including gender, age, tumor location, tumor differentiation, and size (Table 2; Figures 4(a) and 4(b)).

### 3.5. Correlation between ADAMTS8 Methylation and Clinicopathological Factors in Gastric Cancer

We also examined the relationship between ADAMTS8 methylation and the clinicopathologic features of the patients including age, gender, size, differentiation, depth of tumor invasion, and lymph node metastasis. A significant association was found between ADAMTS8 methylation status and lymph node metastasis. There was more frequent methylation in primary tumors with lymph node metastasis than those without lymph node metastasis (71.3 versus 35.0%, *P* = 0.005). For invasive depth, only 40.9% of tumors showed hypermethylation of ADAMTS8 in T1 and T2, compared with T3 and T4 (70.5%) (Table 3).

## 4. Discussion

ADAMTS8 was first discovered together with ADAMTS1 as a novel endogenous angiogenesis inhibitor [22]. To the best of our knowledge, this is the first study to reveal that ADAMTS8 possesses antitumor properties with epigenetic mechanisms underlying its role in gastric cancer. In our current work, we found that ADAMTS8 was downregulated in gastric cancer cell lines compared to normal gastric cells. ADAMTS8 expression was lower in the gastric cancer cell lines SGC7901, MCG803, BGC823, and MKN45 compared to the normal gastric mucosa cell line GES1. We also found that ADAMTS8 expression was lower in gastric cancer tissues but broadly expressed in the nontumor tissues. This study provides evidence that ADAMTS8 may function as a tumor suppressor in gastric cancer. Previous studies have reported that downregulated ADAMTS8 expression in lung cancer and breast cancer did not correlate with clinicopathological factors [20, 30]. In our current study, we found that gastric cancer with lymph node metastases showed significantly lower expression of ADAMTS8. In addition, tumor with invasive depth at T3 and T4 had much lower expression than that with invasive depth at T1 and T2. The result might indicate a possible association between ADAMTS8 and invasiveness of gastric cancer.

Epigenetic silencing of genes by aberrant DNA methylation is recognized as a crucial component of the mechanism underlying tumorigenesis. Accumulating evidence has suggested that the hypermethylation of tumor suppressor genes promoter is one of the major molecular alterations in cancer development. It has been reported that ADAMTS8 expression was epigenetically silenced in brain cancer [23], non-small-cell lung cancer [20], and thyroid cancer [27]. Dunn et al. [23] reported that 1 of 24 brain tumors and 3 of 4 glioma cell lines showed promoter methylation using MSP. Dunn et al. [20] also reported abnormal hypermethylation of ADAMTS8 in 67% of lung adenocarcinomas and 50% of lung squamous cell carcinomas, but the methylation status of ADAMTS8 promoter did not correlate with age, differentiation, or TNM status. We analyzed the methylation status of ADAMTS8 in gastric cancer cell lines, gastric cancer tissues, and corresponding nontumor tissues by MSP and BGS. We found that ADAMTS8 was hypermethylated in MKN45 cells, partially methylated in BGC823 and MGC803 cells, and not methylated in SGC7901 and GES1 cells. We further found that methylation of the ADAMTS8 gene was significantly higher in primary gastric tumors than in nontumor tissues. These data indicate that DNA methylation may play an important role in ADAMTS8 expression in gastric cancer cells and tissues. Furthermore, lower levels of ADAMTS8 mRNA in gastric cancer closely correlate with higher levels of gene methylation. In addition, our results showed that a significant association was found between ADAMTS8 methylation status and lymph node metastasis. There was more frequent methylation in primary tumors with lymph node metastasis than those without lymph node metastasis. We also found that only 40.9% of tumors showed hypermethylation of ADAMTS8 in T1 and T2, compared with T3 and T4 (70.5%). The result might indicate a possible association between DNA methylation of ADAMTS8 and invasion and metastasis of gastric cancer.

To confirm hypermethylation-mediated ADAMTS8 silencing in gastric cancer, we treated gastric cancer cell lines with ADC, a demethylating agent inhibitor, and found that ADAMTS8 expression could be restored via demethylation treatment with ADC in gastric cancer cell lines but not unmethylated SGC7901 cells. This suggests that reactivation of ADAMTS8 correlates with decreased DNA methylation. However, an understanding of the precise mechanism of the downregulation of ADAMTS8 in gastric cancer needs to be further explored. Taken together, these findings suggest that hypermethylation may be an important mechanism in the inactivation of ADAMTS8 in gastric cancer.

In conclusion, our results demonstrate that expression of ADAMTS8 is significantly decreased and DNA methylation is frequent in gastric cancer. ADAMTS8 hypermethylation is associated with decreased expression in gastric cancer and may play an important role in the invasion and metastasis of gastric cancer. However, further research focusing on the epigenetic silencing mechanism underlying the role of ADAMTS8 in gastric cancer is required.

## Figures and Tables

**Table 1:** Methylation status of ADAMTS8 between T and N.

Group	Case	Methylation (%)	No methylation (%)	*P* value
T	66	40 (60.6)	26 (39.4)	
N	66	27 (40.9)	39 (59.1)	0.024^a^

^a^ *P* < 0.05. T: gastric tissues; N: nontumor tissues; ADAMTS8: ADAM metallopeptidase with thrombospondin type 8 motif.

**Table 2:** Correlation of ADAMTS8 mRNA expression and clinicopathological characteristics of gastric cancer samples.

Variable	Patients (*n*)	ADAMTS8 expression relative to GAPDH	*P* value
Age (years)			
<65	40	0.290 ± 0.102	
≥65	26	0.267 ± 0.115	0.405
Gender			
Male	45	0.285 ± 0.112	
Female	21	0.273 ± 0.099	0.674
Tumor differentiation			
Well/moderate	33	0.267 ± 0.111	
Poor	33	0.295 ± 0.103	0.289
Invasive depth			
T1 + T2	22	0.329 ± 0.101	
T3 + T4	44	0.257 ± 0.103	0.009^a^
Tumor location			
Upper + middle	13	0.300 ± 0.110	
Lower	53	0.276 ± 0.107	0.481
Size (cm)			
<3	28	0.287 ± 0.128	
≥3	38	0.277 ± 0.091	0.713
Lymph node metastasis			
No	20	0.402 ± 0.064	
Yes	46	0.229 ± 0.075	<0.001^a^

^a^ *P* < 0.05; ADAMTS8: ADAM metallopeptidase with thrombospondin type 8 motif.

**Table 3:** Clinicopathological characteristics of gastric cancer samples and ADAMTS8 methylation.

Variable	Patients (*n*)	ADAMTS8 methylation		*P* value
		M (%)	U (%)	
Age (years)				
<65	40	25 (62.5)	15 (37.5)	
≥65	26	15 (57.7)	11 (42.3)	0.365
Gender				
Male	45	29 (64.4)	16 (35.6)	
Female	21	11 (52.4)	10 (47.6)	0.219
Tumor differentiation				
Well/moderate	33	23 (69.7)	10 (30.3)	
Poor	33	17 (51.5)	16 (48.5)	0.614
Invasive depth				
T1 + T2	22	10 (40.9)	12 (59.1)	
T3 + T4	44	30 (70.5)	14 (29.5)	0.075
Tumor location				
Upper + middle	13	8 (61.5)	5 (38.5)	
Lower	53	32 (60.4)	21 (39.6)	0.478
Size (cm)				
<3	28	15 (53.6)	13 (46.4)	
≥3	38	25 (65.8)	13 (34.2)	0.621
Lymph node metastasis				
No	20	7 (35.0)	13 (65.0)	
Yes	46	33 (71.3)	13 (28.7)	0.005^a^

^a^ *P* < 0.05. M: methylated and partially methylated cases; U: unmethylated cases; ADAMTS8: ADAM metallopeptidase with thrombospondin type 8 motif.
